# Purple Perilla Extracts Allay ER Stress in Lipid-Laden Macrophages

**DOI:** 10.1371/journal.pone.0110581

**Published:** 2014-10-15

**Authors:** Sin-Hye Park, Daekeun Shin, Soon Sung Lim, Jae-Yong Lee, Young-Hee Kang

**Affiliations:** 1 Department of Food science and Nutrition, Hallym University, Chuncheon, Kangwon-do, Republic of Korea; 2 Department of Biochemistry, Hallym University, Chuncheon, Kangwon-do, Republic of Korea; St. Georges University of London, United Kingdom

## Abstract

There is a growing body of evidence that excess lipids, hypoxic stress and other inflammatory signals can stimulate endoplasmic reticulum (ER) stress in metabolic diseases. However, the pathophysiological importance and the underlying mechanisms of this phenomenon remain unknown. The current study investigated that 50 ng/ml oxidized LDL promoted unfolded protein response (UPR) and ER stress in J774A1 murine macrophages, which was blocked by extracts (PPE) of purple *Perilla frutescens*, a plant of the mint family Lamiaceae. The ER stressor tunicamycin was employed as a positive control. Treating 1–10 µg/ml oxidized LDL for 24 h elicited lipotoxic apoptosis in macrophages with obvious nuclear condensation and DNA fragmentation, which was inhibited by PPE. Tunicamycin and oxidized LDL activated and induced the UPR components of activating transcription factor 6 and ER resident chaperone BiP/Grp78 in temporal manners and such effects were blocked by ≥5 µg/ml PPE. In addition, PPE suppressed the enhanced mRNA transcription and splicing of X-box binding protein 1 (XBP1) by tunicamycin and oxidized LDL. The protein induction and nuclear translocation of XBP1 were deterred in PPE-treated macrophages under ER stress. The induction of ATP-binding cassette transporter A1 (ABCA1), scavenger receptor-B1 (SR-B1) and intracellular adhesion molecule-1 (ICAM-1) was abolished by the ER stressor in activated macrophages. The protein induction of ABCA1 and ICAM1 but not SR-B1 was retrieved by adding 10 µg/ml PPE to cells. These results demonstrate that PPE inhibited lipotoxic apoptosis and demoted the induction and activation of UPR components in macrophages. PPE restored normal proteostasis in activated macrophages oxidized LDL. Therefore, PPE was a potent agent antagonizing macrophage ER stress due to lipotoxic signals associated with atherosclerosis.

## Introduction

An accumulation of unfolded or misfolded proteins in the endoplasmic reticulum (ER) lumen causes a cellular ER stress initiating unfolded protein response (UPR) [1]. The UPR functions in order to primarily restore normal proteostasis in the ER by halting protein translation and activating the signaling pathways leading to the production of molecular chaperones involved in protein folding [2]. In the UPR scenario, transmembrane ER resident kinases including inositol-requiring enzyme 1α (IRE1α) are activated, and membrane-anchored activating transcription factor 6 (ATF6) is monomerized, followed by activation of versatile transcription factors of X-box binding protein 1 (XBP1) [1], [2]. The UPR instigates cell survival by improving protein folding capacity of ER, or promotes cell death following chronic ER stress [3]. Excessive activation of sustained UPR results in cellular dysfunction and cell death as major contributors to cancer and neurodegenerative diseases [4], [5]. Unresolved ER stress is involved in a variety of metabolic disorders, such as obesity and type 2 diabetes mellitus [6].

Emerging preclinical and clinical evidence supports the notion that pharmacological modulators of ER stress have therapeutic potential as novel treatments of metabolic disorders, including obesity, fatty liver disease, and atherosclerosis [7], [8]. The molecular mechanisms of UPR in the pathogenesis of metabolic diseases emphasize the roles of various UPR components in animal disease models and human pathologies [9], [10]. The emergence of compounds that target specific UPR signaling components, such as ATF6, IRE1, spliced XBP1 and the proteasomes, would provide promising therapeutics for the treatment of human metabolic diseases [11], [12]. Chemical chaperones such as sodium phenylbutyrate, reduce ER stress and restore glucose homeostasis in a mouse model of type 2 diabetes [13]. The ER modification by chemical chaperones in macrophages and adipocytes has therapeutic efficacy against atherosclerosis in mouse models [14].

Some clinically approved pharmacological agents used in clinical settings may affect the UPR pathways. The lipid-lowering compound pravastatin ameliorates ER stress in cultured neonatal rat cardiomyocytes and in pressure-overloaded hearts, and the anti-diabetic agent pioglitazone represses hepatic ER stress in reducing insulin resistance [15], [16]. Recent evidence suggests potential benefits from phytochemicals and polyphenols in reducing the elevated oxidative and lipid-mediated ER stress [17], [18]. Vaticanol B, a resveratrol derivative, inhibits inflammation and improves the ER environment by reducing the ER protein load and by maintaining the ER membrane integrity [19]. Additionally, grape seed proanthocyanidin extracts alleviate oxidative stress and ER stress in the skeletal muscle in the pathogenesis of type 2 diabetes mellitus [20]. The phytoalexins glyceollins enhances insulinotropic actions in enteroendocrine cells and normalizes glucose homeostasis through diminishing ER stress [21]. Furthermore, quercetin protects macrophages from oxidized low-density lipoprotein (LDL)-induced apoptosis by inhibiting the ER stress-CHOP signaling pathway [22].

Purple perilla (*Perilla frutescens*) has been used in traditional medicine for respiratory afflictions and seasonal allergies due to its anti-allergic and anti-inflammatory activity [23]. In our previous study [24], purple *perilla frutescens* leaf extract (PPE) inhibited aldose reductase activity. The present study attempted to investigate that PPE inhibited the induction of UPR signaling components in the ER stressor tunicamycin-exposed murine macrophages. This study examined ER stress components and PPE cytoprotection from lipotoxicity in lipid-laden macrophages. Furthermore, it was explored that PPE inhibited ER stress-triggered proteostasis of atherogenesis-associated biomarkers in activated macrophages.

## Materials and Methods

### Materials

Dulbecco’s Modified Eagle Medium (DMEM) chemicals, fatty acid-BSA, tunicamycin, and β-actin antibody were purchased from Sigma Aldrich Chemical (St. Louis, MO), as were all other reagents, unless specifically stated elsewhere. Fetal bovine serum (FBS) and penicillin-streptomycin were obtained from Lonza (Basel, Switzerland). Antibodies of ATF6, BiP/GRP78 and XBP1 were obtained from Novus Biologicals (Littleton, CO). Antibodies of ABCA1, scavenger receptor B1 (SR-B1) and intracellular adhesion molecule-1 (ICAM-1) were provided from Abcam (Cambridge, UK). The liver X receptor (LXR) agonist T091317 was purchased by Cayman Chemical Company (Ann Arbor, MI). HRP-conjugated goat anti-rabbit IgG was supplied from Jackson ImmunoResearch Laboratory (West Grove, PA). 4′,6-Diamidino-2-phenylindole (DAPI) was obtained from Santa Cruz Biotechnology (Santa Cruz, CA).

### Preparation of purple perilla extracts

Purple perilla was purchased from a local market in Chuncheon, Korea. A voucher sample (RIC-2012-5) has been deposited at the Center for Efficacy Assessment and Development of Functional Foods and Drugs, Hallym University, Chuncheon, Korea. The specimens were authenticated by Emeritus Professor Hyung-Joon Chi, Seoul National University, Korea. Dried leaves of purple perilla frutescens (2 kg) were extracted 3 times with 99.5% methanol for 5 h. The solvent was evaporated under reduced pressure below 45°C to give a methanol extract (11.68% yields). The extract was suspended in distilled water and partitioned with n-hexane (n-Hex), methylene chloride (CH_2_Cl_3_), ethyl acetate (EtOAc), n-butanol (n-BuOH), and H_2_O to yield 40.83 g n-Hex (purple perilla extract, PPE), 25.20 g EtOAc, 22.24 g CH_2_Cl_3_, 116.88 g n-BuOH, and 27.42 g H_2_O fractions [24].

### Preparation and oxidation of human plasma LDL

Human plasma LDL was prepared by a discontinuous density gradient ultra-centrifugation as previously described [25], [26]. The plasma LDL isolation was approved by the Hallym University Institutional Review Board (HIRB-2011-007-2). The written informed consent was waived by the HIRB for use of blood samples. A pooled human normolipidemic plasma LDL fraction was dialyzed and used within 4 weeks. The concentration of total cholesterol was measured by using commercial diagnostic kits (Asan Pharmaceuticals, Seoul, Korea). Oxidized LDL was prepared by incubating with 10 µM CuSO_4_ in F-10 medium at 37°C for 24 h. The extent of LDL oxidative modification was routinely determined using TBARS measurements and eletrophoretic mobility assay.

### Macrophage culture and viability

Mouse (BALB/cN, ascites) macrophage-like cell line J774A1 (American Type Culture Collection, Manassas, VA) were grown in DMEM supplemented with 10% FBS at 37°C in a humidified atmosphere of 5% CO_2_ in air [26]. Macrophages were pre-treated with 1–10 µg/ml PPE and exposed to 50 µg/ml cholesterol-oxidized LDL or 1 µM tunicamycin for various times. J774A1 macrophages were incubated in DMEM supplemented with 0.4% fatty acid-free BSA and treated with CuSO_4_-oxidized LDL.

The cytotoxicity of 1 µM tunicamycin was determined using MTT assay after culture of J774A1 macrophages in the presence of PPE. J774A1 cells were incubated in a fresh medium containing 1 mg/ml MTT for 3 h at 37°C. The purple formazan product was dissolved in 0.5 ml isopropanol with gentle shaking. Absorbance of formed formazan was measured at λ = 570 nm using a microplate reader (Bio-Rad Model 550, Hercules, CA). In the current study PPE *per se* did not induce toxicity of cells within the concentration range of 1–10 µg/ml (data not shown).

### DNA laddering

The DNA laddering assay was conducted with a commercial apoptotic DNA ladder detection kit (invitrogen, Camarillo, CA), according to the manufacture’s instruction. Cells were treated with 1 µM tunicamycin or 10 µg/ml PPE and extracted in a Tris-EDTA lysis buffer. Ammonium acetate solution and 100% ethanol were added for the precipitation of DNA debris at –20°C. After centrifugation at 12,000 rpm for 10 min, DNA pellets were washed with 70% ethanol and air-dried for 10 min. Dried DNA pellets were re-suspended by adding DNA suspension buffer containing Tris, glycerol, and orange G and then electrophoresed on 1% agarose gel with 0.5 µg/ml ethidium bromide. For the visualization of DNA ladders, gel images were taken by TFX-20 M model-UV transilluminator (Vilber-Lourmat, Marne-la-Vallée, France).

### Hoechst 33258 staining

After treating 10 µg/ml PPE to macrophages exposed to 1 µM tunicamycin on a glass-chamber slide, a brief washing with PBS-0.2% Tween 20 was conducted. Cells were fixed with 4% formaldehyde for 15 min. After blocking cells with a 4% FBS for 1 h, cells were stained with 1 mg/ml of the nuclear dye Hoechst 33258 (Promega Co., Madison, WI) to visualize nuclear condensation and fragmentation under fluorescence microscopy. Images of each slide were taken using an optical microscope system (Axiomager, Zeiss, Oberkochen, Germany).

### Terminal deoxynucleotidyl transferase dUTP nick end labeling (TUNEL) assay

For the measurement of cellular apoptosis and DNA fragmentation, TUNEL assay was conducted by using commercial Florometric TUNEL kits (Promega Co.). J774A1 murine macrophages were incubated with 1 µM tunicamycin for 24 h in the absence and presence of 10 µg/ml PPE and fixed with 4% formaldehyde 4°C for 25 min. Fixed cells were permeabilized with 0.2% Triton X-100 and labeled fragmented DNA with fluorescein-dUTP at 37°C for 1 h. DAPI was used for counter-staining nuclei that were visualized and quantified with using an Axiomager optical microscope system.

### Western blot analysis

Following culture protocols, cells were lysed in a lysis buffer. Equal protein amounts of cell lysates were electrophoresed on 6–8% SDS-PAGE and transferred onto a nitrocellulose membrane. After blocking with either 5% non-fat dry skim milk or 3% fatty acid-free bovine serum albumin or for 3 h, membrane was incubated overnight at 4°C with polyclonal rabbit antibodies of ATF6, BiP/GRP78, XBP1, ABCA1, SR-B1and ICAM-1. After triple washes, membrane was incubated for 1 h with a goat anti-rabbit IgG conjugated to horseradish peroxidase. The individual protein level was determined using Immobilon Western Chemiluminescnet horseradish peroxidase substrate (Millipore, Billerica, MA). Incubation with mouse β-actin antibody was also performed for comparative controls. After performing immunoblotting, the blot bands were visualized on Agfa X-ray film (Agfa-Gevaert, Belgium).

### RT-PCR analysis

Total RNA was obtained from J774A1 cells using a commercial Trizol reagent kit (Invitrogen, Carlsbad, CA). The cDNA was synthesized using 5 µg of total RNA with 200 units of reverse transcriptase and 0.5 mg/ml oligo-(dT)_15_ primer (Bioneer, Daejeon, Korea). The PCR was accomplished using mRNA transcripts of mouse spliced XBP1 (forward primer: 5′-TACGGGAGAAAACTCACGGC-3′, reverse primer: 5′-TTCCAGCTTGGCTGATGAGG-3′, 421 bp) and glyceraldehyde-3-phosphate dehydrogenase (GAPDH, forward primer: 5′-AACTTTGGCATTGTGGAAGGG-3′, reverse primer: 5′-GACACATTG GGGGTAGGAACAC-3′, 224 bp) with an addition of 25 µl of 10 mM Tris-HCl (pH 9.0) containing 25 mM MgCl_2_, 10 mM dNTP and 5 units of Taq DNA polymerase. XBP1 was conducted 27 cycles and each cycle consisted of 30 s at 94°C, 30 s at 60°C and 45 s at 72°C, and the final extension was for 10 min at 72°C. After thermocycling and electrophoresis of the PCR products (10 µl) on 3% agarose gel containing 0.5 mg/ml ethidium bromide, the bands were visualized using a Vilber-Lourmat UV transilluminator and gel photographs were obtained. The absence of contaminants was routinely checked by the RT-PCR assay of negative control samples without a primer addition.

### Immunocytochemical analysis

Immunofluorescent cytochemical staining for monolayer cells of J774A1 cells grown on glass coverslips was performed using mouse spliced XBP1 antibody and Cy3-conjugated anti-rabbit IgG. Cells were fixed with 4% formaldehyde for 15 min and permeated with 0.1% Triton X-100 for 1 min on ice. Cells were blocked using a 20% FBS for 1 h, and anti-mouse XBP1 was applied to cells. Triple washing was followed and incubation with Cy3-conjugated goat anti-rabbit IgG was achieved for 1 h. Nuclear staining was carried out with 4 mg/ml DAPI. Each slide was mounted in a mounting medium (Sigma Aldrich Chemical). Images of each slide were taken using an optical microscope system. The spliced XBP1 levels were quantified by an image analysis program from the microscope system.

### Data analysis

The results are presented as means ± SEM. Statistical analyses were conducted using the SAS software package version 6.12 (SAS Institute, Cary, NC). One-way ANOVA was used to determine inhibitory effects of PPE on cellular effects of oxidized LDL in macrophages. Differences among treatment groups were analyzed with Duncan’s multiple-range test and considered significant at P<0.05.

## Results

### Suppressive effect of PPE on ER stress-induced apoptosis

**Figure 1:**
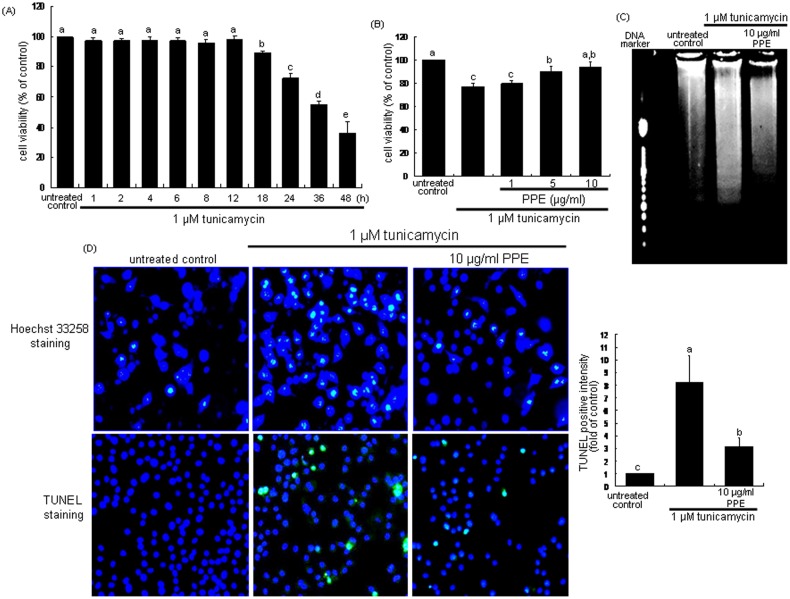
Time course response of cytotoxicity by tunicamycin (A), elevation of cell viability by PPE (B), and DNA laddering (C) and inhibition of nuclear condensation and DNA fragmentation (D) and by PPE in tunicamycin-treated macrophages. MTT assay was performed for the measurement of cell survival of 1 µM tunicamycin-exposed J774A1 murine macrophages by 1–10 µg/ml PPE (A and B). The bar graph data represent mean ± SEM from 4 independent experiments with multiple estimations. Values are expressed as percent cell survival relative to untreated control cells (cell viability = 100%). For the measurement of cellular apoptosis, DNA laddering, Hoechst33258 staining and TUNEL assay (C and D) were conducted with light microscopy and fluorescent microscopy, respectively. Histograms showing the relative fluorescent staining intensity of TUNEL-positive cells (D). Magnification: 200-fold. Means without a common letter differ, P<0.05.

To examine the macrophage death by the ER stressor tunicamycin, MTT analysis was performed. When J774A1 macrophages were incubated with 1 µM tunicamycin for 48 h, ≈30% of macrophages were dead for 24 h (Figure 1A). In contrast, the decreased cell viability was near-completely restored by treating 10 µg/ml PPE (Figure 1B). The DNA laddering was obvious in tunicamycin-exposed macrophages, which was blunted by PPE (Figure 1C). In addition, tunicamycin-induced ER stress caused nuclear condensation and fragmentation of macrophages, while such condensation and fragmentation disappeared in 10 µg/ml PPE-treated cells, evidenced by Hoechst 33258 staining and TUNEL (Figure 1D). Accordingly, PPE alleviated macrophage apoptosis induced by ER stress.

### Blockade of ATF6 activation and BiP/GRP78 induction by PPE

**Figure 2:**
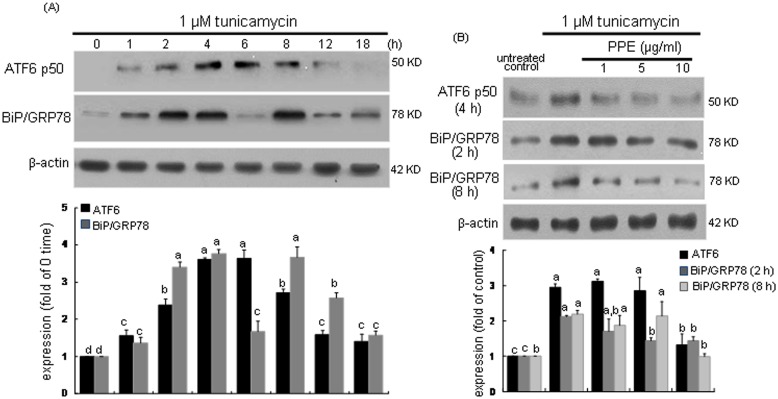
Time course responses of ATF6 and BiP/GRP78 induction (A) and their inhibition by 1–10 µg/ml PPE (B) in 1 µM tunicamycin-treated macrophages. For the measurement of induction of ATF6 and BiP/GRP78 (B), total cell lysates were subject to Western blot analysis with a primary antibody against ATF6 or BiP/GRP78. β-Actin was used as an internal control. The bar graphs (mean ± SEM, n = 3) represent quantitative densitometric results of upper bands. Means without a common letter differ, P<0.05.

This study investigated whether PPE was able to inhibit the induction of UPR components and then alleviate macrophages ER stress. When macrophages were exposed to 1 µM tunicamycin, the ER transmembrane transcription factor ATF6 and the molecular chaperone BiP/GRP78 were temporally induced. As shown in Figure 2A, tunicamycin initiated ATF6 induction from 2 h after its exposure, and the induction was sustained up to 8 h. The ATF6 induction by treating tunicamycin for 6 h was dose-dependently suppressed by ≥5 µg/ml PPE (Figure 2B). On the other hand, tunicamycin also promoted the BiP/GRP78 induction in a dual fashion, in which early induction of BiP/GRP78 occurred from 1 h after the addition of tunicamycin (Figure 2A). In addition, its late induction ensued from 8 h-post exposure. PPE at the doses of ≥5 µg/ml suppressed both early (2 h elapsed) and late (8 h elapsed) inductions of BiP/GRP78 by tunicamycin (Figure 2B).

### Inhibition of XBP1 induction by PPE

**Figure 3:**
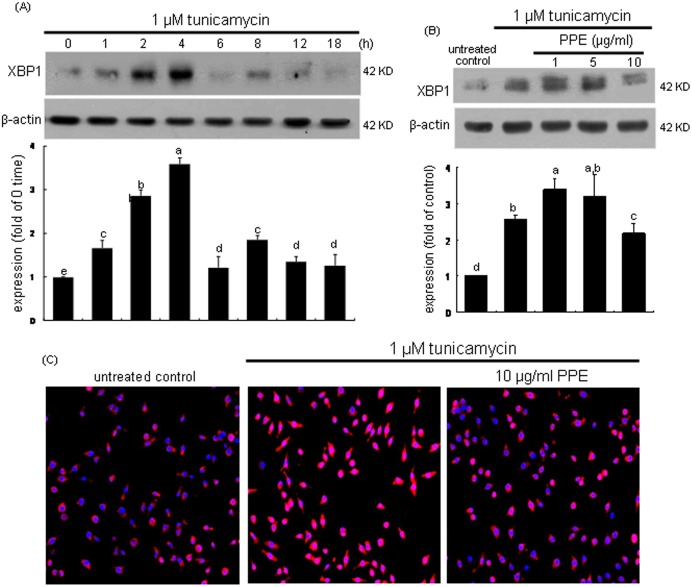
Time course response of XBP1 induction (A) and its inhibition by 1–10 µg/ml PPE (B) and nuclear translocation (C) in 1 µM tunicamycin-exposed macrophages. For the measurement of XBP1 induction, total cell lysates were subject to Western blot analysis with a primary antibody against XBP1. β-Actin was used as an internal control. The bar graphs (mean ± SEM, n = 3) represent quantitative densitometric results of upper bands. Means without a common letter differ, P<0.05. Nuclear translocation of XBP1 was detected by immunofluorocytochemical staining with Cy3-conjugated XBP1 antibody and nuclear counter-staining was carried out with DAPI (C). Microscopic observation was done by fluorescent microscopy. Magnification: 200-fold.

**Figure 4:**
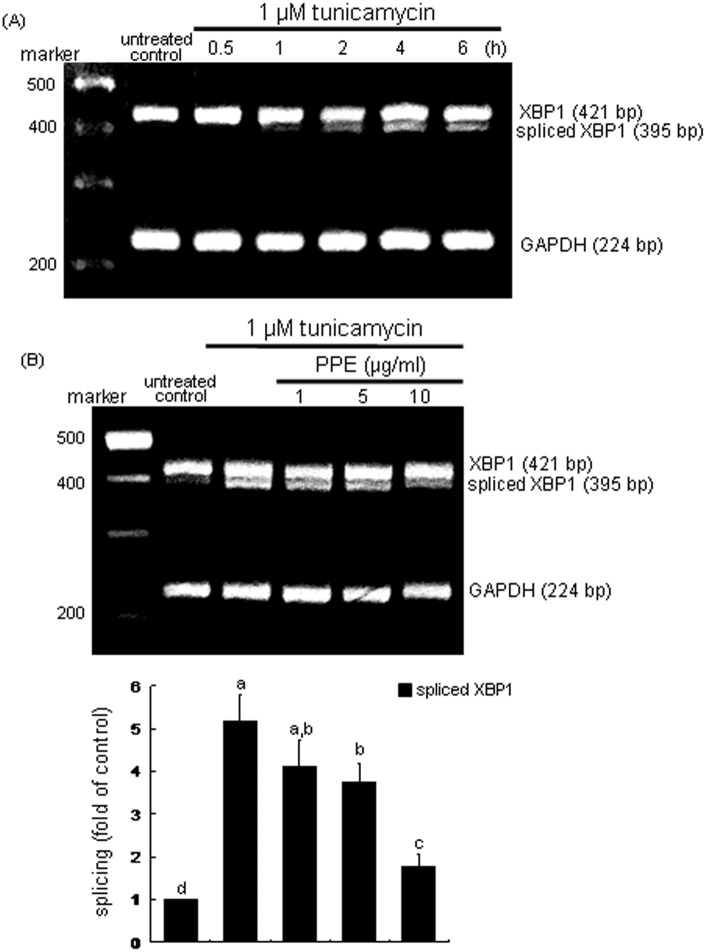
Temporal induction of XBP1 transcription (A) and inhibition of XBP1 transcription (B) by PPE. J774A.1 macrophages were treated with 1 µM tunicamycin for 6 h in the absence and presence of 1–10 µg/ml PPE. XBP1 mRNA levels were measured by quantitative RT-PCR assay (A and B). GAPDH was used for the internal control (n = 3). The bar graphs (mean ± SEM, n = 3) represent quantitative densitometric results of upper bands. Means without a common letter differ, P<0.05.

The transcription factor XBP-1 of macrophages was induced in an early stage within 2 h by tunicamycin (Figure 3A). The XBP1 induction was demoted by treating macrophages with 10 µg/ml PPE (Figure 3B). The nuclear translocation of XBP1 (pinkish staining) was enhanced in tunicamycin-exposed cells, which was deterred by administrating 10 µg/ml PPE (Figure 3C). These results indicates that PPE halted the formation of activated XBP1 leading to promoted transcriptional activation of UPR components. When the ER stress occurs, XBP1 mRNA is spliced by the endoRNase IRE1 and translocated into nucleus [1], [2]. As shown in Figure 4A, the XBP1 mRNA splicing was induced from 2 h after tunicamycin insult and such induction was sustained for another 2 h. In PPE-treated cells, the XBP-1 mRNA remained unspliced (Figure 4B). Accordingly, tunicamycin-triggered activation and nuclear translocation of the transcription factor XBP1, which was disturbed by adding PPE to macrophages.

### Repressive effect of PPE on oxidized LDL Induction of ER stress

**Figure 5:**
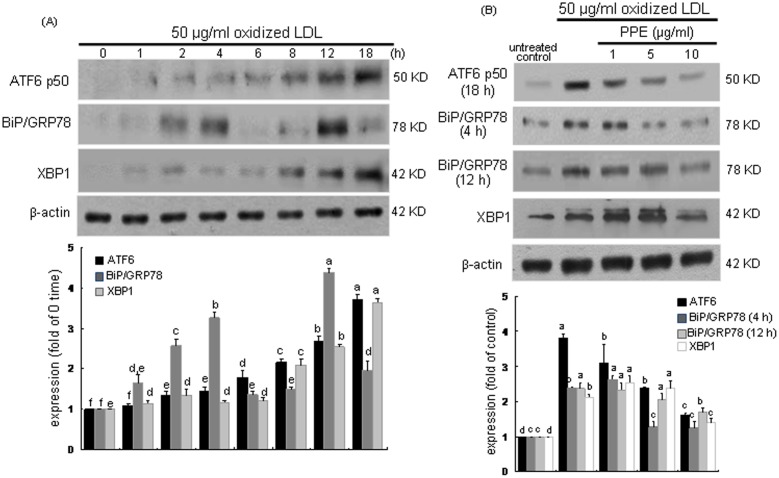
Time course responses of ATF6, BiP/GRP78 and XBP1 (A) and their inhibition by 1–10 µg/ml PPE (B) in 1 µM tunicamycin-treated macrophages. For the measurement of induction of ATF6, BiP/GRP78 and XBP1 (B), total cell lysates were subject to Western blot analysis with a primary antibody against ATF6, BiP/GRP78 or XBP1. β-Actin was used as an internal control. The bar graphs (mean ± SEM, n = 3) represent quantitative densitometric results of upper bands. Means without a common letter differ, P<0.05.

Oxidized LDL is a critical factor in atherogenesis by triggering activation of inflammatory signaling pathway that may contribute to endothelial activation and dysfunction [27]. This study examined whether oxidized LDL elicited ER stress like tunicamycin and PPE inhibited ER stress in lipid-laden macrophages. Temporal induction of the ER stress markers of ATF6, BiP/GRP78 and XBP1 by oxidized LDL was observed (Figure 5A). Western blot analysis revealed that ATF6 activation and XBP1 expression were enhanced from 8 h after the exposure to 50 µg/ml oxidized LDL. The BiP/GRP78 induction was also enhanced in a dual manner, where oxidized LDL induced the BiP/GRP78 expression from 2 h to 4 h and at 12 h after its exposure (Figure 5A). It should be noted that oxidized LDL induced the respective UPR components later than tunicamycin did. When 10 µg/ml PPE was added to oxidized LDL-experienced macrophages, the enhanced ATF6 activation and XBP1 induction were significantly attenuated (Figure 5B). In addition, PPE abolished the dual induction of BiP/GRP78. Therefore, PPE blocked oxidized LDL-elicited ER stress via up-regulating the activation and induction of the UPR components of ATF6, BiP/GRP78 and XBP1.

**Figure 6:**
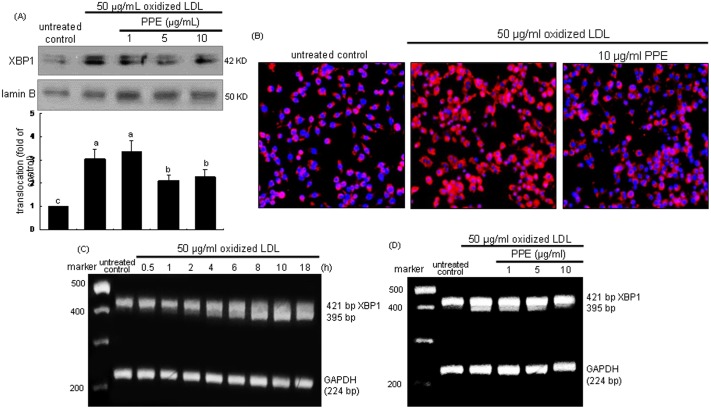
Inhibition of nuclear translocation (A and B), and temporal response of XBP1 transcription and its inhibition by PPE (C and D). J774A.1 macrophages were treated with 50 µg/ml oxidized LDL for 18 h in the absence and presence of 1–10 µg/ml PPE. Nuclear extracts were isolated by nuclear fraction assay and Western blot analysis was conducted by using primary anti-XBP1 (A). Lamin B was used as a nuclear control. Values (mean ± SEM, n = 3) not sharing a common letter are different at P<0.05. Nuclear translocation of XBP1 was also detected by immunofluorocytochemical staining with Cy3-conjugated XBP1 antibody and nuclear counter-staining was carried out with DAPI (B). Microscopic observation was done by fluorescent microscopy. Magnification: 200-fold. XBP1 mRNA levels were measured by quantitative RT-PCR assay (C and D). GAPDH was used for the internal control (n = 3).

As shown in Figure 6A, nuclear XBP1 protein level was enhanced by treating cells with 50 µg/ml oxidized LDL, which was repressed by ≥5 µg/ml PPE. Consistently, the oxidized LDL-triggered nuclear translocation of XBP1 was disturbed in 10 µg/ml PPE-administrated macrophages, evidenced by Cy3-immunofluorocytochemical staining (Figure 6B). The XBP1 mRNA splicing was noticeably observed from 4 h after exposure to oxidized LDL (Figure 6C). In contrast, the spliced XBP-1 mRNA level was diminished in 1–10 µg/ml PPE-treated cells (Figure 6D). Thus, PPE was effective in inhibiting the transcriptional activity of XBP1 in lipid-laden macrophages.

### Restoration of protein folding activity by PPE

**Figure 7:**
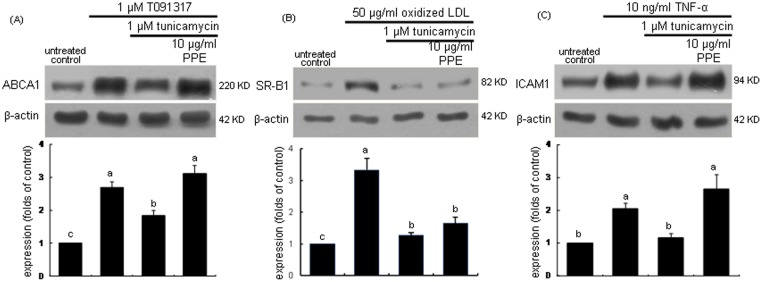
PPE restoration of induction of ABCA1(A), SR-B1 (B) and ICAM-1 (C) demoted in tunicamycin-treated J774A1 murine macrophages. Cells were stimulated with 1 µM T091317 for 12 h, 50 µg/ml Cu^2+^-oxidized LDL for 6 h, and 10 ng/ml TNF-α for 6 h in the absence and presence of 1 µM tunicamycin and 10 µg/ml PPE. For the measurement of expression of ABCA1, SR-B1 and ICAM-1, total cell lysates were subject to Western blot analysis with a primary antibody against ABCA1, SR-B1 and ICAM-1. β-Actin was used as an internal control. The bar graphs (mean ± SEM, n = 3) represent quantitative densitometric results of upper bands. Means without a common letter differ, P<0.05. Values (mean ± SEM, n = 3) not sharing a common letter are different at P<0.05.

This study examined whether PPE retrieved the protein folding activity impaired by ER stress in macrophages. The LXR agonist T091317 at 1 µM, 50 µg/ml oxidized LDL and 10 ng/ml tumor necrosis factor (TNF)-α were employed for the macrophage induction of ABCA1, SR-B1, and ICAM1, respectively. These proteins were significantly stimulated by the respective stimulators (Figure 7). When the stimulated cells were exposed to 1 µM tunicamycin, the induction of ABCA1, SR-B1, and ICAM1 was abolished to their basal levels. In contrast, the treatment with 10 µg/ml PPE regained the induction of ABCA1 and ICAM1. These results demonstrate that PPE recovered the protein folding capacity of macrophage ER for the induction of ABCA1 and ICAM1. In contrast, the protein induction of SR-B1 by oxidized LDL was still impaired in 10 µg/ml PPE-treated macrophages, indicating that PPE did not affect the SR-B1 folding activity inhibited by the ER stressor.

## Discussion

Seven major findings were observed from this study. 1) J774A.1 macrophages incubated with 1 µM tunicamycin for 24 h were dead by ≈30%, which was reversed by treating ≥5 µg/ml PPE. 2) PPE alleviated the ER stressor-induced apoptosis of macrophages with the concomitant inhibition of nuclear condensation and DNA laddering. 3) The PPE treatment ameliorated ER stress by reducing the activation and induction of UPR components of ATF6 and BiP/GRP78. 4) Tunicamycin induced XBP1 splicing and activation in macrophages, which was disturbed by administrating PPE to cells. 5) Oxidized LDL caused macrophage ER stress slowly with temporal activation and induction of ATF6, BiP/GRP78 and XBP1. 6) PPE ameliorated the ER stress in lipid-laden macrophages by demoting the activation and induction of these components. 7) Tunicamycin suppressed the protein induction of ABCA1 and ICAM1 in activated macrophages, whereas PPE retrieved the induction of these proteins. These observations demonstrate that PPE encumbered apoptosis and lipotoxicity, and restored normal proteostasis in ER stress-experienced macrophages. Therefore, PPE would be a promising candidate alleviating ER stress in macrophages involved in atherogenesis.

Cellular stimuli such as oxidative insult and calcium disturbance lead to the accumulation of unfolded proteins, a condition referred to as ER stress [1], [4]. In this study oxidized LDL containing oxysterols resulted in ER stress as the ER stress enhancer tunicamycin. Oxidized LDL highly activated the transmembrane transcription factor ATF6 and the transcription factor XBP1 in macrophages. The UPR increases the production of ER-resident chaperones triggering protein folding signaling pathways [1]. Oxidized LDL like tunicamycin induced the molecular chaperone BiP/GRP78 in a dual fashion. If excessive UPR in the ER of macrophages is sustained, cells will experience apoptotic cell death. Consistently, the nuclear condensation and DNA fragmentation appeared in macrophages treated with tunicamycin for 24 h. Since ER stress is involved in a variety of metabolic disorders [7], the therapeutic interventions targeting against UPR components and reducing ER stress would be promising strategies to treat metabolic diseases [11], [12]. Several studies demonstrate that phytochemicals and polyphenols reduce oxidative and lipid-mediated ER stress, hence inhibiting inflammation, obesity, and atherosclerosis [17]–[20]. Grape seed proanthocyanidins alleviate oxidative stress and skeletal muscle ER stress in the pathogenesis of type 2 diabetes mellitus [20]. In the present study PPE suppressed ER stress-induced macrophage apoptosis by tunicamycin through disturbing the induction and activation of UPR components. ER stress pertaining to the induction of small molecular chemical chaperones is a highly promising therapeutic target for metabolic diseases.

The balance between protein load and protein folding capacity of ER influences cell survival [3]. There is a growing body of evidence that a key cellular event in vulnerable atherosclerotic plaques is ER stress-induced macrophage apoptosis [12], [14], [28]. The mechanisms for the transition between adaptation to ER stress and ER stress-induced cell death are still being understood. The current study revealed that tunicamycin suppressed the protein synthesis of ABCA1, SR-B1 and ICAM-1 in activated macrophages, all involved in HDL biogenesis or atherogenesis. The protein ABCA1 is known as the cholesterol efflux regulatory protein and is a major regulator of cellular cholesterol and phospholipid homeostasis [29]. PPE restored the ABCA1 folding activity demoted by ER stress, suggesting that PPE may improve HDL biogenesis. Although the ICAM-1 induction in activated macrophages declined under ER stress, PPE also retrieved its protein expression and restored normal proteostasis. However, PPE did not improve SR-B1 expression deterred by ER stress in lipid-laden macrophages. The protein SR-B1 regulates the uptake of HDL-derived cholesterol and cholesteryl ester in steroidogenic tissues and facilitates macrophage cholesterol flux that may partly account for its effects on atherogenesis [30], [31]. It cannot be ruled out that PPE *per se* may attenuate SR-B1 induction by oxidized LDL despite its alleviation of ER stress.

The activation of these UPR signaling pathways plays a pro-survival role in the early stage of atherosclerosis [32]. With the progression of atherosclerosis, the increased intensity of ER stress in atherosclerotic lesions lead to prolonged and enhanced UPR signaling. UPR pathways induce expression of death effectors and activate apoptosis signaling pathways, leading to apoptosis of macrophages and smooth muscle cells in advanced lesions [32]. Prolonged oxidized LDL induced macrophage ER stress and apoptosis with the up-regulation of UPR components. Macrophages in atherosclerotic plaques produce abundant secretory proteins via UPR signaling pathways, which potentially induce ER stress in these cells [33]. Therapeutic approaches of PPE targeting the UPR may have promise in the prevention and/or regression of atherosclerosis. Similarly, quercetin protects macrophages from oxidized LDL-induced apoptosis by inhibiting the ER stress-CHOP signaling pathway [22].

Macrophage apoptosis can be caused by the accumulation of free cholesterol in the ER, leading to activation of the UPR [34], [35]. It is deemed that oxysterols present in oxidized LDL were the predominant regulator of oxidized LDL-induced macrophage ER stress. PPE promoted cholesterol efflux from macrophages through restoring the ABCA1 induction, possibly inhibiting lipotoxic apoptosis. In addition, PPE can encumber the uptake of oxidized lipids, which may render macrophages resistant to oxidized LDL-induced ER stress. The SR-B1 induction was not enhanced in PPE-administrated macrophages under ER stress.

In summary, the current study demonstrated that PPE reduced ER stress-induced apoptosis and alleviated UPR in J774A1 murine macrophages. Oxidized LDL promoted the induction of ER transcription factors and molecular chaperones, which was suppressed by PPE. In addition, PPE enhanced the induction of ABCA1 and ICAM1 demoted due to ER stress in activated macrophages. Accordingly, PPE encumbered lipotoxic apoptosis and restored normal proteostasis under ER stress of macrophages. Although PPE may serve as a beneficial in vitro modulator targeting against ER stress, the in vivo role of PPE remains unclear.

## Supporting Information

File S1 **Hallym University Institutional Review Board (HIRB-2011-007-2) for the plasma LDL isolation.**
(PDF)

File S2 **Certification for the exemption of written consent for use of blood samples.**
(PDF)
