# Supplementary material for: Purple Perilla Extracts Allay ER Stress in Lipid-Laden Macrophages
Source: PLoS One. 2014 Oct 15;9(10):e110581. doi: 10.1371/journal.pone.0110581 (PMC4198214; doi:10.1371/journal.pone.0110581)
Supplement: File S1 — Hallym University Institutional Review Board (HIRB-2011-007-2) for the plasma LDL isolation. (PDF) [file pone.0110581.s001.pdf]

한림대학교 생명윤리심의위원회 심사결과 통지서
NOTIFICATION FROM HALLYM UNIVERSITY INSTITUTIONAL REVIEW BOARD (HIRB)

(200-702) 강원도 춘천시 한림대학길 39 한림대학교 연구처 연구지원팀
Research Section of Hallym University, 39 Hallymdaehak-gil, Chuncheon-si, Gangwon-do, 200-702 Korea.
Tel : +82-33-248-1020~8, Fax : +82-33-248-1029 E-mail: de1020@hallym.ac.kr

연구책임자<br>Director of Research	소속<br>Institution	한림대학교 자연과학대학 식품영양학과		
	직위<br>Position	교수		
	성명<br>Name	강영희		
심의의뢰자<br>Review Client	소속<br>Institution	한림대학교 자연과학대학 식품영양학과		
	직위<br>position	석사과정생	본 과제내 역할<br>Roles in this Research	실험자
	성명<br>Name	박신혜		
심의분야<br>Field of Review	☐ 유전자 연구 (Genetic Research)<br>☐ 유전자 검사 (Genetic Examination)<br>☑ 기타 (Others) : 혈액 내 LDL분리<br>연구계획의 법적·윤리적 적합성			
연구방법<br>Method of Research	☐ 설문조사(Survey) ☑ 검체채취(Sample-gathering)<br>☐ 검체분석(Sample-analysis) ☑ 검체보관(Sample-storge)<br>☐ 검체이관(Sample-transfer)<br>☐ 기타(Othres)			
연구 과제명<br>Title of Research	LDL에 의해 유도된 죽상동맥경화증에서 효과를 가지는 식물성 소재의 탐색			
연구비 지원기관<br>Provider of Research Fund	지역거점연구단 육성사업			
연구기간 및 심의 유효기간<br>Dates of Research & Available Period of Decision from HIRB	부터(FROM)	2011년 8월 2일		
	까지(UP TO)	2014년 8월 2일		
심의 결과<br>Decision from HIRB	☑ 승인 (Approval)<br>☐ 조건부승인 (Conditional Approval)<br>☐ 부결 (Rejection)<br>※ If 'Director of Research' would like to file a protest against HIRB, please contact the Research Section, Hallym University, at the above-mentioned address within 15 days.			
심의일자<br>Date of Decision	2011년 9월 8일			
심의번호<br>IRB Number	HIRB-2011-007-2			
기타<br>Remarks				

상기와 같이 결정되었음을 통보합니다.

The above-referenced protocol has been reviewed by the Hallym University Institutional Review Board (HIRB). The above-mentioned proposed research has been approved in accordance with HIRB bylaws.

2011년 9월 8일 (September 8, 2011)

한림대학교 생명윤리심의위원회 위원장
CHAIRMAN OF INSTITUTIONAL REVIEW BOARD, HALLYM UNIVERSITY
