# Supplementary material for: Purple Perilla Extracts Allay ER Stress in Lipid-Laden Macrophages
Source: PLoS One. 2014 Oct 15;9(10):e110581. doi: 10.1371/journal.pone.0110581 (PMC4198214; doi:10.1371/journal.pone.0110581)
Supplement: File S2 — Certification for the exemption of written consent for use of blood samples. (PDF) [file pone.0110581.s002.pdf]

동의서 면제 확인서

(Certification for exemption of written consent)

► 연구명 **(Title of Research)** : LDL에 의해 유도된 죽상동맥경화증에서 효과를 가지는 식물성 소재의 탐색

► 면제 이유 **(The reason of Exemption)**

: 본 연구를 위해 검체로서 혈액을 필요하는 바입니다. 그러나 병원에서 타인의 검체를 제공받아 사용하는 것이 아니라 본 연구에 참여하는 실험자의 혈액을 직접 채취할 것입니다. 본 연구에 참여하는 실험자인 박신혜, 김민수 연구원의 혈액을 채취하는 것이므로 검체의 채취를 위한 동의서는 불필요하다고 사료됩니다. 즉, 병원에서 타인의 혈액 검체를 제공받는 것이 아니므로 타인에게 검체 제공 동의서를 작성하는 것은 무의미하다고 판단됩니다. 따라서 박신혜, 김민수 연구원의 혈액 채취를 위해 검체제공 동의서를 직접 작성해 진행할 수도 있지만 검체의 제공자와 제공받는 자가 동일하기 때문에 검체제공 동의서의 면제를 신청하는 바입니다. 동의서의 면제를 신청하지만 검체를 제공하는 데 있어서 생명윤리위원회의 규칙에 전적으로 따를 것이며, 혈액 잔여물의 폐기여부도 적법한 절차를 걸쳐 처리할 것입니다.

상기 내용을 확인하고 동의서 면제 내용을 승인합니다.

The above-mentioned the reason for the exemption of written consent has been reviewed by the Hallym University Institutional Review Board (HIRB). The exemption of the written consent has been approved in accordance with HIRB bylaws.

2014년 6월 13일 (June 13, 2014)

한림대학교 생명윤리심의위원회 위원장

CHAIRMAN OF INSTITUTIONAL REVIEW BOARD, HALLYM UNIVERSITY
